# Supplementary material for: (-)-Englerin-A Has Analgesic and Anti-Inflammatory Effects Independent of TRPC4 and 5
Source: Int J Mol Sci. 2021 Jun 15;22(12):6380. doi: 10.3390/ijms22126380 (PMC8232259; doi:10.3390/ijms22126380)
Supplement: Supplementary file 1 [file ijms-22-06380-s001.zip › ijms-1243075-supplementary.pdf]

International Journal of *Molecular Sciences*

MDPI

Supplementary material

Table S1. Summary of mechanical paw withdrawal thresholds measured at baseline and at 2 and 4 hours after intra-plantar injections of carrageenan in the left hindpaw.

	Vehicle		EA (2 mg/kg)			EA (4 mg/kg)		
Time (h)	Mean ± SEM	vs baseline	Mean ± SEM	vs baseline	vs vehicle	Mean ± SEM	vs baseline	vs vehicle
0	0.88±0.07	-	0.94±0.02	-	>.999	0.98±0.02	-	1.00
2	0.19±0.07	<.001	0.42±0.14	0.00	0.58	0.68±0.10	0.28	0.01
4	0.11±0.02	<.001	0.25±0.11	<.001	0.96	0.96±0.55	0.03	0.02

Table S2. Summary of thermal paw withdrawal thresholds measured at baseline and at 1 and 3 hours after intra-plantar injections of carrageenan in the left hindpaw.

	Vehicle		EA (2 mg/kg)			EA (4 mg/kg)		
**Time (h)**	Mean ± SEM	**vs baseline**	Mean ± SEM	**vs baseline**	**vs vehicle**	Mean ± SEM	**vs baseline**	**vs vehicle**
0	9.48±0.98	-	8.01±0.38	-	0.86	8.61±1.44	-	0.99
1	5.84±0.98	0.04	6.97±1.32	0.98	0.96	9.84±0.93	0.94	0.02
3	4.83±0.81	0.00	6.26±0.92	0.71	0.88	4.79±0.29	0.02	>0.99

Table S3. Summary of thermal paw withdrawal thresholds measured at baseline and at 1 and 3 hours after intra-plantar injections of carrageenan in the left hindpaw.

	Vehicle		EA (2 mg/kg)		
Time (h)	Mean ± SEM	vs baseline	Mean ± SEM	vs baseline	vs vehicle
0	7.98±0.06	-	7.82±0.07	-	0.9949
1	4.46±0.40	<0.0001	4.71±0.32	<0.0001	0.9696
3	3.58±0.23	<0.0001	6.41±0.18	0.0154	<0.0001

Supplementary table 4: Summary of mechanical paw withdrawal thresholds measured at baseline and at 2 and 4 hours after intra-plantar injections of carrageenan in the left hindpaw.

	WT Vehicle		WT EA			TRPC5 KO Vehicle			TRPC5 KO EA		
Time (h)	Mean ± SEM	vs baseline	Mean ± SEM	vs baseline	vs vehicle	Mean ± SEM	vs baseline	vs vehicle	Mean ± SEM	vs baseline	vs vehicle
0	1.00±0.01	-	0.98±0.02	-	>.999	0.99±0.01	-	>.999	0.98±0.01	-	>.999
2	0.46±0.11	<.001	0.98±0.02	>.999	<.001	0.43±0.09	<.001	>.999	0.87±0.06	0.972	0.001
4	0.20±0.04	<.001	0.60±0.13	0.003	0.002	0.22±0.07	<.001	>.999	0.88±0.06	0.992	<.001

Supplementary table 5: Summary of thermal paw withdrawal thresholds measured at baseline and at 1 and 3 hours after intra-plantar injections of carrageenan in the left hindpaw.

	WT Vehicle		WT EA			TRPC5 KO Vehicle			TRPC5 KO EA		
Time (h)	Mean ± SEM	vs baseline	Mean ± SEM	vs baseline	vs vehicle	Mean ± SEM	vs baseline	vs vehicle	Mean ± SEM	vs baseline	vs vehicle
0	9.70±0.73	-	8.58±0.47	-	1.00	9.50±0.93	-	>0.9999	8.32±0.99	-	1.00
2	5.45±1.14	0.11	9.26±1.42	>0.9999	0.21	7.11±0.82	0.83	0.98	9.21±1.13	>0.9999	0.23
4	4.75±0.95	0.03	7.15±0.98	1.00	0.82	4.28±0.59	0.04	>0.9999	6.85±1.70	0.99	0.92

Supplementary table 6: Summary of mechanical paw withdrawal thresholds measured at baseline and at 2 and 4 hours after intra-plantar injections of carrageenan in the left hindpaw.

	Veh+Veh		Veh+EA			ML204+Veh			ML204+EA		
Time (h)	Mean ± SEM	vs baseline	Mean ± SEM	vs baseline	vs vehicle	Mean ± SEM	vs baseline	vs vehicle	Mean ± SEM	vs baseline	vs vehicle
0	0.99±0.01	-	0.96±0.04	-	>.999	0.98±0.04	-	>.999	0.96±0.03	-	>.999
2	0.38±0.17	<.001	0.86±0.11	1.00	0.00	0.53±0.07	0.01	0.95	0.72±0.05	0.45	0.11
4	0.28±0.06	<.001	0.79±0.12	0.89	0.00	0.28±0.05	<.001	>.999	0.54±0.11	0.02	0.40

Supplementary table 7: Summary of thermal paw withdrawal thresholds measured at baseline and at 1 and 3 hours after intra-plantar injections of carrageenan in the left hindpaw.

	Veh+Veh		Veh+EA			ML204+Veh			ML204+EA		
Time (h)	Mean ± SEM	vs baseline	Mean ± SEM	vs baseline	vs vehicle	Mean ± SEM	vs baseline	vs vehicle	Mean ± SEM	vs baseline	vs vehicle
0	8.19±0.74	-	7.67±1.06	-	>0.9999	9.37±2.58	-	>0.9999	6.86±0.84	-	1.00
2	6.83±0.65	1.00	10.69±1.89	0.88	0.62	5.77±0.89	0.71	>0.9999	11.41±1.23	0.40	0.39
4	4.45±1.24	0.67	8.04±1.72	>0.9999	0.72	4.34±1.47	0.26	>0.9999	7.00±3.07	>0.9999	0.96

Supplementary table 8: Summary of dose-response curve of cultured primary sensory neurons from WT and KO mice to increasing concentration of Englerin-A.

Englerin-A concentration (nM)	WT	TRPC5 KO
0.1	0.00	0.00
0.3	10.04±6.17	3.67±9.17
1	21.20±8.26	23.81±0.39
3	30.49±15.98.17	26.66±11.87
10	53.48±1.90	52.12±3.80
30	79.80±9.00	85.07±6.86
100	76.41±6.50	81.75±15.64
